# Building a Nomogram for Metabolic Syndrome Using Logistic Regression with a Complex Sample—A Study with 39,991,680 Cases

**DOI:** 10.3390/healthcare10020372

**Published:** 2022-02-14

**Authors:** Min-Seok Shin, Jea-Young Lee

**Affiliations:** Department of Statistics, Yeungnam University, Gyeongsan 38541, Korea; minseok3830@gmail.com

**Keywords:** logistic regression, metabolic syndrome, nomogram, risk factor

## Abstract

Metabolic syndrome can cause complications, such as stroke and cardiovascular disease. We aimed to propose a nomogram that visualizes and predicts the probability of metabolic syndrome occurrence after identifying risk factors related to metabolic syndrome for prevention and recognition. We created a nomogram related to metabolic syndrome in this paper for the first time. We analyzed data from the Korea National Health and Nutrition Examination Survey VII. Total 17,584 participants were included in this study, and the weighted sample population was 39,991,680, which was 98.1% of the actual Korean population in 2018. We identified 14 risk factors affecting metabolic syndrome using the Rao-Scott chi-squared test. Next, logistic regression analysis was performed to build a model for metabolic syndrome and 11 risk factors were finally obtained, including BMI, marriage, employment, education, age, stroke, sex, income, smoking, family history and age* sex. A nomogram was constructed to predict the occurrence of metabolic syndrome using these risk factors. Finally, the nomogram was verified using a receiver operating characteristic curve (ROC) and a calibration plot.

## 1. Introduction

Metabolic syndrome is a disease that includes conditions such as obesity, hyperlipidemia, low levels of high-density lipoprotein (HDL) cholesterol, high blood pressure, and hyperglycemia coincide in one individual due to chronic metabolic disorders. Metabolic syndrome was first named Syndrome X in 1988 [1]; however, in 1999, the World Health Organization (WHO) renamed it as metabolic syndrome. The WHO definition of metabolic syndrome has not been consistently used because of its requirement to measure serum insulin and urinary microalbumin levels [2,3]. Therefore, we used the diagnostic criteria for metabolic syndrome published by the National Cholesterol Education Program [4]. Metabolic syndrome is diagnosed when three or more of the following criteria are met: obesity (waist circumference ≥ 90 cm for men; waist circumference ≥ 85 cm for women), hyperlipidemia (triglyceride levels ≥ 150 mg/dL), low levels of high-density lipoprotein cholesterol (men < 40 mg/dL; women < 50 mg/dL), high blood pressure (systolic BP ≥ 130 mmHg and diastolic BP ≥ 85 mg/dL or if a patient is on hypertension medication), and hyperglycemia (fasting glucose ≥ 100 mg/dL). In the United States, 32.8 and 36.6% of male and female individuals aged 20 years or older, respectively, were diagnosed with metabolic syndrome in 2012 [5]. In Korea, approximately 30.8 and 26.3% of male and female individuals over 20 years of age, respectively, were diagnosed with metabolic syndrome in 2013 [6]. Furthermore, 28.1% of men and 18.7% of women were diagnosed with metabolic syndromes in 2017 [7]. Since metabolic syndrome causes complications, such as stroke and cardiovascular diseases, identifying strategies to prevent metabolic syndrome occurrence is essential [8].

The Pearson chi-squared test is a statistical analysis method used to identify risk factors for diseases. Logistic regression and Cox proportional hazards models are statistical models used to predict disease incidence. Many studies have been conducted to identify the risk factors for metabolic syndrome; however, interpreting and understanding the results is challenging for medical practitioners and individuals without statistical knowledge. Therefore, we aimed to develop a nomogram to compensate for this limitation.

A nomogram graphically represents the numerical relationships between diseases and risk factors without the need for complex calculations [9,10]. Nomograms have previously been developed for dyslipidemia and hypertension [11,12]. Although many studies have identified risk factors for metabolic syndrome, there has been no attempt to create a nomogram for metabolic syndrome. Now, this study, we used the Rao-Scott chi-squared test instead of the Pearson chi-squared test to identify risk factors from the Korean National Health and Nutrition Examination Survey (KNHANES) data, and after constructing a multinomial logistic regression model, we constructed a nomogram that can predict the incidence rates of metabolic syndrome. Finally, the constructed nomogram was verified using an ROC curve and a calibration plot.

Section 2 describes the materials used in this study and the Rao–Scott chi-squared test. Next, we explain how to build and verify a nomogram using the calculated coefficient from the multinomial logistic regression of complex sampling data. In Section 3, we present the results of the Rao-Scott chi-squared test and the logistic regression analysis. Additionlly, the nomogram for metabolic syndrome was constructed and verified. Finally, Section 4 and Section 5 present the discussion and conclusions of this study.

## 2. Materials and Methods

### 2.1. Complex Sampling Design Method

Data collected using a simple random sampling method revealed that each element had the same probability of being selected and was independent of the others. However, the KNHANES data used in this study were designed to sample representative the Korean population using census data as a sampling frame and a two-stage stratified cluster sampling method. Complex sample data consider the design effect involving stratification, clustering, and individual sample weight corrected for inclusion error, imbalanced extraction rate, and non-response error of the target population.

### 2.2. Materials

The 2016–2018 data used in this study were obtained from KNHANES. KNHANES uses a two-stage stratified cluster sampling method, which is a complex sampling design method, to improve the estimation accuracy and representativeness of the sample [13]. This study was conducted with subjects aged 20 years or older. In 3 years, 5072 of the 24,269 individuals under 20 years of age were excluded. Further, 1613 individuals who did not participate in the health or examination surveys were excluded. The sample population then comprised of 17,584 individuals. The mode was used to replace the missing values. We also compared the complex sample population with the actual Korean population surveyed in the 2018 census [14]. The weighted sample population was 39,991,680. According to the Statistics Korea 2018 census, 40,762,796 Korean individuals were older than 20 years of age. The difference between the complex sample and the actual Korean population was 1.9% of the actual population. Therefore, the weighted sample and actual Korean population were considered to be similar. The data were divided into a training set and a test set using a ratio of 8:2. Thus, the weighted sample population of the training set was 32,043,914 and that of the test set was 7,947,766. The model was fitted using the training set and its predictive power was verified using the test set.

The criteria for the diagnosis of metabolic syndrome included three or more of the following factors; abdominal obesity, hyperlipidemia, low HDL cholesterol, hypertension, and hyperglycemia. We used the metabolic syndrome diagnostic criteria published by the National Cholesterol Education Program (NCEP ATP III) in 2001. However, among the five diagnostic criteria, abdominal obesity was applied by the Korean Society for the Study of Obesity in 2014 to reflect the characteristics of Koreans. In this study, 4850 of 17,584 individuals were diagnosed with metabolic syndrome.

Fourteen risk factors with an important effect on the incidence of diabetes, dyslipidemia, and hypertension have been selected in several prior studies [12,15,16,17]. These risk factors include body mass index (BMI), Marriage, Employment, Education, Age, Stroke, Sex, Income, Heart attack, Exercise, Alcohol, Angina, Smoking, and Family history. BMI was categorized into three groups: <25, ≥25 and <30, and ≥30 [12]. Participants were categorized as “low” for individuals who had not completed high-school, and “high” for those who had completed high-school education. Age was categorized as 20–34 years, 35–64 years, and ≥65 years. Participants were categorized into lowest, low, high, and highest groups based on income. Regarding exercise status, participants were classified into the physical activity group if they walked for more than 5 days per week for more than 30 min walking a day. Participants were categorized into the high drinking group if they drank more than once a month in the past year or in the low drinking group if otherwise. Smoking habits were categorized as present, past, and nonsmoking. The other variables were categorized as yes or no.

### 2.3. Statistical Analysis

#### 2.3.1. Rao-Scott $\chi_{Rao-Scott}^{2}$ Test

It is important to test the independence between the incidence of metabolic syndrome and the risk factors when selecting risk factors that affect metabolic syndrome incidence. The Pearson chi-squared test has been used to identify the risk factors for metabolic syndrome. However, this test assumes that the frequency of each cell in the contingency table is independent and follows a multinomial distribution. However, the data used in this study are complex data, given different individual weights for each stratum and cluster. Therefore, it does not satisfy the assumption that each cell in the table is independent [12,18]. Therefore, the Rao-Scott chi-squared test, which considers the design effects, such as stratification, clustering, and individual sample weight, was used in this study.

#### 2.3.2. Logistic Regression Model with a Complex Sample

When estimating the regression coefficients and variance of the coefficient, logistic regression analysis of a complex sample considers design effects such as stratification, clustering, and individual weights [19,20,21]. First, the population was divided into *K* strata, and each stratum was divided into $M_{k}$ clusters. The first unit of collection divided by the stratum is called the primary sample unit (PSU). Each cluster had $i=1,2,\ldots ,N_{kj}$ observations. Thus, supposing that $n_{kj}$ observations exist in each PSU selected for each *k* stratum. The total number of observations was $n=\sum_{k=1}^{K}\sum_{j=1}^{M_{k}}n_{kj}$. The weight of each observation is denoted as $\omega_{kji}$. This study assumed that the dependent variable $y_{kji}$ is a binary variable. $y_{kji}$ represents the binomial value of the *i*-th observation value in the *j*-th PSU within the *k*-th stratum. $X_{kji}={\left(x_{kji1},x_{kji2},\ldots ,x_{kjip}\right)}^{{}^{\prime }}$ represents the independent variable vector of the *i*-th observation value in the *j*-th PSU within the *k*-th stratum. We added the interaction terms by considering the association between the independent variables. Let a be the number of interaction terms. $\beta ={\left(\beta_{0},\beta_{1},\ldots ,\beta_{p},\beta_{p+1},\ldots ,\beta_{p+a}\right)}^{{}^{\prime }}$ is a $\left(p+a+1\right)\times 1$ vector indicating the regression coefficient vector of the logistic model. The logistic regression model was as follows:

$$\begin{array}{cc} \; & \ln\frac{P\left(y_{kji}=1|x_{kji}\right)}{1-P\left(y_{kji}=1|x_{kji}\right)}\\ & =\beta_{0}+\beta_{1}X_{1}+\beta_{2}X_{2}+\cdot \cdot \cdot +\beta_{p}X_{p}+\beta_{p+1}X_{1}X_{2}+\cdot \cdot \cdot +\beta_{p+a}X_{p-1}X_{p} \end{array}$$

The maximum pseudo-likelihood method was used to estimate the regression coefficient vector β for the complex sample. $\omega_{kji}$ denotes the weight of each observation in the logistic regression model.

#### 2.3.3. Nomogram Construction Method

Several medical studies have aimed to predict methods for reduce the risk of disease or death. Statistical techniques are used to select the relevant disease or death risk factors and to calculate the extent of their effects for risk prediction. Logistic regression analysis is the most widely used method in medical research; however, it is difficult to interpret. Therefore, a nomogram was proposed to estimate the probability of an event [9,22]. A nomogram is easily understood because of its simplified building process, and a line easily expresses its composition. A nomogram comprises the point, risk factor, probability, and total point lines. The construction of the lines that form the nomogram has been explained previously explained [9,23,24].

(a) Point line: A point line comprising 0–100 points is constructed.

(b) Risk factor line: The $LP_{ij}\left(LinearPredictor\right)$ value is calculated from the coefficient of the fitted logistic regression model. If the independent variable *X* is a categorical variable and has *j* categories, j−1 dummy variables are generated.

$$LP_{ij}=\beta_{ij}\times X_{ij}$$

Using this, we calculated $Points_{ij}$ for each risk category and aligned them to each risk factor line.

$$Points_{ij}=\frac{LP_{ij}-\min_{j}LP_{ij}}{\max_{j}LP_{*j}-\min_{j}LP_{*j}}\times 100$$

In this case, $\beta_{ij}$ is the regression coefficient value of the *j*-th category of the *i*-th risk factor and $X_{ij}$ is the attribute value of the *j*-th category of the *i*-th risk factor. $LP_{*j}$ represents the LP value of the risk factor with the largest estimated regression coefficient range of attribute values.

(c) Probability line: The probability line represents the probability value corresponding to the total point and ranges from 0 to 1.

(d) Total point line: The total point can be expressed as a cumulative sum of $Points_{ij}$.

$$Total\;Points=\sum_{i,j}Points_{ij}=\frac{100}{\max_{j}LP_{*j}-\min_{j}LP_{*j}}\times \sum_{i,j}\left(LP_{ij}-\min_{j}LP_{ij}\right)$$

The logistic regression model is expressed for $\sum_{i,j}LP_{ij}$, and by substituting this into the above equation, the total points corresponding to each value of the probability line can be obtained.

$$\begin{array}{cc} Total\;Points= & \frac{100}{\max_{j}LP_{*j}-\min_{j}LP_{*j}}\times \\ & \left(\ln\left(\frac{P\left(Y=1|X=x\right)}{1-P\left(Y=1|X=x\right)}\right)-\alpha -\sum_{i,j}\min_{j}LP_{ij}\right) \end{array}$$

The value of the probability line is substituted for $P\left(Y=1|X=x\right)$ to construct a total point line.

#### 2.3.4. ROC Curve and Calibration Plot for Nomogram Validation

After constructing the nomogram, ROC curves and calibration plots were used to validate the nomogram [25,26]. 1-specificity and Sensitivity was plotted on the *x*-axis and *y*-axis, respectively. The AUC (Area Under the Curve) with a diagonal line was 0.5. The model is considered good when the ROC curve is above this diagonal line, and better when the value is between 0.5 and 1. A calibration plot was used to determine the closeness of the actual probabilities to the predicted probabilities calculated using the nomogram. If the predicted probability was the same as the actual probability, a 45° centerline was drawn. The closer the predicted probability is to the actual probability, the closer it is to the 45° line [9,27]. Therefore, we validated the nomogram using $R^{2}$, a goodness-of-fit indicator of the regression line between the predicted and actual probabilities. All analyses were performed using R software version 4.1.2 (R Core Team, Vienna, Austria). We also used SAS 9.4 to build a nomogram with the suitable aesthetic [24].

## 3. Results

### 3.1. 14 Risk Factors Associated Metabolic Syndrome by Rao-Scott Chi-Squared Test

We used the Rao–Scott chi-squared test to select risk factors related to metabolic syndrome. Table 1 shows the weighted frequency and results of the Rao-Scott chi-squared test. As shown in Table 1, the prevalence of metabolic syndrome increased with increasing BMI. For example, the incidence of metabolic syndrome was 12.1, 45.1, and 66.4% for BMI < 25, ≥25 and <30, and ≥30, respectively. Additionally, older individuals had a higher incidence of metabolic syndrome. The incidence rates were 8.2%, 26.4%, and 45.1% for 20–34, 35–64, and ≥65 years, respectively. Further, 41.6% and 19.9% of individuals from the low and high education level categories were diagnosed with metabolic syndrome, respectively. The incidence rates were 29.0% and 10.7% for married and unmarried individuals, respectively. Moreover, 54.2% of patients diagnosed with stroke and 24.5% of those not diagnosed with stroke developed metabolic syndrome. The incidence rates in the non-smoking, past smoking, and present smoking groups were 21.7%, 29.2%, and 28.9%, respectively. Men and women had metabolic syndrome incidence rates of 28.3% and 21.5%, respectively. Furthermore, 45.1% and 24.7% of patients diagnosed with and without angina developed metabolic syndrome, respectively. The lower the income, the higher was the incidence of metabolic syndrome. Individuals with metabolic syndrome were generally unemployed, inactive, with a history of heart attack, low drinkers, and with a family history of metabolic syndrome. The results of the Rao-Scott chi-squared test $\left(\chi_{R-S}^{2}\right)$ showed that all factors were statistically significant at 0.05. Therefore, 14 risk factors were found to be important for predicting the incidence of metabolic syndrome.

### 3.2. Multiple Logistic Results for Metabolic Syndrome

A logistic regression analysis was performed using the risk factors listed in Table 1. The results, which only considered the main effects of 14 risk factors, showed that the regression coefficients for Heart attack, Exercise, Alcohol, and Angina were not significant. Thus, Heart attack, Exercise, Alcohol, and Angina had no significant effect on the prediction of metabolic syndrome. Therefore, ten risk factors were selected as final risk factors for metabolic syndrome. Further, the interactions among the risk factors were considered to improve the predictive power, and the BMI*Marriage, Marriage*Age, Age*Sex, and Sex*Smoking interactions were significant. However, a model including the ten main effects and the interaction of Age and Sex was selected as the final logistic regression model after considering the parsimony of the model and the likelihood ratio, Wald, and score test results. The likelihood ratio, Wald, and score tests were performed to assess the goodness-of-fit of the logistic regression model, and the results showed that the *p*-value was less than 0.0001. Table 2 shows the logistic regression results with the ten main effects and one interaction. A “95% CI” represents the 95% confidence interval of the odds ratio, and “$Point_{ij}$” indicates the nomogram point for each category of risk factors. Among the risk factors, BMI had the greatest effect on the incidence of metabolic syndrome (Table 2). Further, the incidence of metabolic syndrome was increased in older males with a history of stroke. The interaction variable showed that male sex aged 35–64 years had the greatest impact on metabolic syndrome, whereas male sex and age over 65 years had the smallest impact because of the lower incidence of metabolic syndrome in males aged ≥ 65 years than in women. The interaction variable complements the odds ratios for age and sex.

### 3.3. 11 Risk Factors with a Proposed Nomogram for Metabolic Syndrome

**Figure 1:**
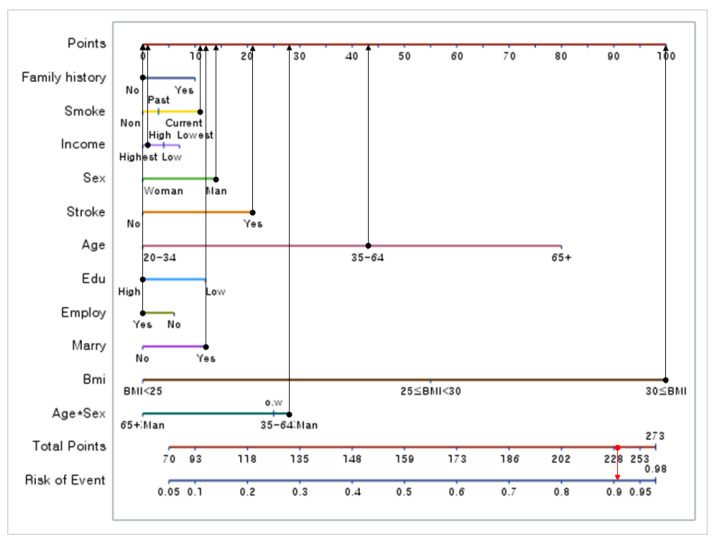
Nomogram result for metabolic syndrome by 11 risk factors.

A nomogram was constructed using the logistic regression model (Table 2). In Figure 1, a nomogram is proposed to predict the prevalence of metabolic syndrome. Figure 1 shows that BMI and Age had the greatest impact on metabolic syndrome. The higher the BMI, the higher is the incidence of metabolic syndrome. In addition, the incidence of metabolic syndrome increased with age. After BMI and Age, Age*Sex had the greatest impact, followed by Stroke, excluding BMI, Age, and Age*Sex. Individuals with a history of stroke had higher scores than those without; therefore, the former were more likely to have metabolic syndrome. Subsequently, metabolic syndrome was influenced by Marriage, followed by Education, Smoking, Family history, Income, and Employment. After multiple logistic regression analysis, the nomogram model for metabolic syndrome consisted of 11 risk factors. The result was shown in Figure 1. For example, if a 50-year-old man with a BMI of 30 is married, employed, a college graduate, has a history of stroke, belongs to a high-income level, a smoker, and without a family history, he would obtain nomogram points as follows: 100, 12, 0, 0, 43, 21, 14, 28, 1, 11, and 0 points for BMI, Marriage, Employ, Education, Age, Stroke, Sex, Age*Sex, Income, Smoke, and family history lines, respectively, leading to a summed score of 230. The probability of metabolic syndrome corresponding to the total number of points is 90.7%.

### 3.4. Validation of a Nomogram for Metabolic Syndrome

**Figure 2:**
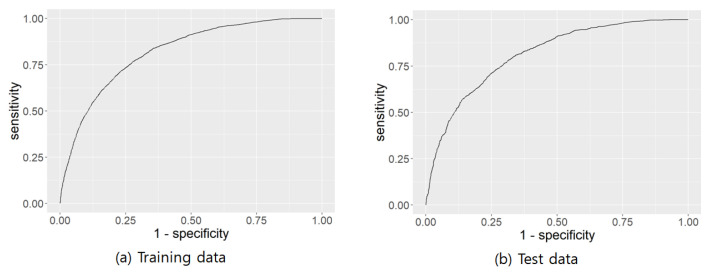
ROC curve validation of a nomogram for metabolic syndrome.

**Figure 3:**
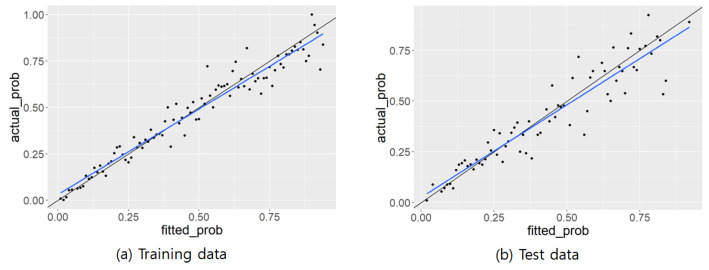
Calibration plot of nomogram for metabolic syndrome.

The nomogram was verified using an ROC curve and a calibration plot. The results are shown in Figure 2 and Figure 3, respectively. Figure 2a shows the ROC curve of the training data, and Figure 2b shows the ROC curve of the test data. The AUC (Area Under the Curve) values were 0.8205 and 0.8123 for the training and test data ROC curves, respectively. Figure 3a shows the calibration plot of the training data and Figure 3b shows the calibration plot of the test data. The $R^{2}$ values for the calibration plots were 0.945 and 0.8762, respectively. Therefore, it can be concluded that the nomogram had sufficient predictive power.

## 4. Discussion

This study used data from 2016 to 2018 for 17,584 individuals from the KNHANES, which identifying the health behavior of Koreans. However, when the individual weights assigned to each stratum and cluster were applied, the complex sample population was 39,991,680, which is 98.1% of the actual Korean population surveyed in the 2018 Korean Census [14]. Thus, a complex sample analysis is more reasonable than a raw data analysis. The Rao-Scott chi-squared test was thus used to screen for risk factors of metabolic syndrome, and all 14 risk factors were found to be statistically significant. In the results of multiple logistic regression analysis considering the main effects of 14 above, a total of 11 factors were finally selected: BMI, Marriage, Employment, Education, Age, Stroke, Sex, Income, Smoking, Family history and Age*Sex. Based on the above results, Heart attack, Exercise, Alcohol, and Angina were not significant. They were important as single factors (Table 1), but because of their relatively small chi-square statistics (Heart attack = 26.427, Exercise = 29.7132, Alcohol = 24.9774, Angina = 45.1976), they were not selected in the logistic regression analysis. In particular, in the case of exercise variable, people who walked for more than 5 days a week and more than 30 min a day were defined as physical activity groups, and the boundary between physical activity and non-physical activity was considered somewhat ambiguous. In this case, the Rao Scott chi-squared test statistic value = 29.7132 was not large, and the logistic analysis did not appear to be significant.

Analysis of the model considering all interactions of risk factors showed that BMI*Marriage, Marriage*Age, Age*Sex, and Sex*Smoke were significant. However, considering the parsimony of the model and the statistics of the likelihood ratio and the Wald test and score test results, the interaction between age and sex was included. Therefore, ten main effects and the Age*Sex interaction were finally selected as a total 11 risk factors for metabolic syndrome.

Meanwhile, BMI, Age, Sex, Marriage, Education, Smoking, Income, and Employment were similar compared to previous studies for metabolic syndrome [6,8]. However, in our study, Stroke and Family history were newly identified risk factors. Influence of Stroke was also after BMI, Age, and Age*Sex (Figure 1). Nomogram studies on dyslipidemia [11], hypertension [12] and diabetes [15] showed that BMI, Employment, Education, Age, Sex, Income, and Smoking were common significant factors. On the other hand, the Stroke factor was not present in the studies of dyslipidemia [11] and diabetes [15] but was found to be a significant factor in the study of metabolic syndrome.

Nomogram was composed of risk factors including BMI, Marriage, Employment, Education, Age, Stroke, Sex, Income, Smoking, Family history, and Age*Sex (Figure 1). As shown in Figure 1, the risk factors for metabolic syndrome were important in the order of BMI, Age, Age*Sex, Sex, and Stroke. In other words, the higher the BMI, the greater was the effect on incidence. In addition, the older the patient are, the higher was the prevalence of metabolic syndrome. Age*Sex was also important. Further, men were found to be more likely to have metabolic syndrome than women. Furthermore, a history of stroke, lower educational level, smoking history, family history of metabolic disease, lower income level, and unemployment increase the risk of developing metabolic syndrome. The accuracy of the proposed nomogram was evaluated using the area under the curve (AUC) and $R^{2}$ of the calibration plot. In the training data ROC curve, the AUC was 0.8205, whereas, it was 0.8123, in the test data ROC curve. The $R^{2}$ values of the calibration plot were 0.945 and 0.8762 for the training and test datasets, respectively. In other words, the proposed nomogram had sufficient reliability. However, if the interaction term is added to the logistic model, interpretation may be somewhat difficult [21]. This also occurs in the nomogram model. Meanwhile, the nomogram model has a great advantage in that it shows the influence of risk factors when diagnosing metabolic syndrome as a score [10].

## 5. Conclusions

Metabolic syndrome increases in incidence and is a fatal cause of stroke, cardiovascular disease, and increased death rate. We used 39,991,680 individuals, a complex sample population constituting 98.1% of the actual Korean population surveyed in the 2018 Korean census. In the proposed nomogram (Figure 1) for predicting the incidence of metabolic syndrome, the most relevant risk factor for metabolic syndrome was BMI, followed by Age, Sex, Stroke, Marriage, Education, Smoking, Family history, Income, and Employment. A nomogram facilitates medical practitioners to diagnose a disease. Thus, the metabolic syndrome nomogram proposed in this study will assist in establishing future medical treatment plans.

## Figures and Tables

**Table 1:** Rao-Scott chi-squared test result for 14 risk factors associated metabolic syndrome.

Variable	Level	Metabolic (%)	Non-Metabolic (%)	$\chi_{R-S}^{2}$	*p*-Value
BMI	BMI < 25	3,138,741 (12.1)	22,748,807 (87.9)	2128.3904	0.0001
	25 ≤ BMI < 30	5,365,781 (45.1)	6,542,466 (54.9)		
	30 ≤ BMI	1,458,791 (66.4)	737,094 (33.6)		
Marriage	Yes	9,003,536 (29.0)	22,056,181 (71)	309.0303	0.0001
	No	959,776 (10.7)	7,972,187 (89.3)		
Employment	Yes	6,182,962 (23.4)	20,207,289 (76.6)	29.727	0.0001
	No	3,780,350 (27.8)	9,821,079 (72.2)		
Education	Low	3,844,170 (41.6)	5,393,007 (58.4)	942.6711	0.0001
	High	6,119,142 (19.9)	24,635,361 (80.1)		
Age	20–34	815,925 (8.2)	9,186,929 (91.8)	1006.4028	0.0001
	35–64	6,192,295 (26.4)	17,245,747 (73.6)		
	65+	2,955,092 (45.1)	3,595,692 (54.9)		
Stroke	Yes	283,695 (54.2)	240,097 (45.8)	91.1563	0.0001
	No	9,679,617 (24.5)	29,788,271 (75.5)		
Sex	Man	5,684,391 (28.3)	14,390,899 (71.7)	87.496	0.0001
	Woman	4,278,921 (21.5)	15,637,469 (78.5)		
Income	Lowest	2,875,053 (28.4)	7,264,837 (71.6)	38.88	0.0001
	Low	2,571,634 (25.5)	7,519,515 (74.5)		
	High	2,367,455 (23.7)	7,637,714 (76.3)		
	Highest	2,149,170 (22.0)	7,606,302 (78.0)		
Heart attack	Yes	130,116 (45.5)	155,918 (54.5)	26.427	0.0001
	No	9,833,196 (24.8)	29,872,450 (75.2)		
Exercise	Physical activity	3,319,421 (22.1)	11,682,340(77.9)	29.7132	0.0001
	Non-physical activity	6,643,892 (26.6)	18,346,028 (73.4)		
Alcohol	High drink	5,543,185 (23.4)	18,150,203 (76.6)	24.9774	0.0001
	Low drink	4,420,127 (27.1)	11,878,165 (72.9)		
Angina	Yes	217,589 (45.1)	265,396 (54.9)	45.1976	0.0001
	No	9,745,723 (24.7)	29,762,972 (75.3)		
Smoking	Non	4,878,365 (21.7)	17,595,872 (78.3)	88.4268	0.0001
	Past	2,569,051 (29.2)	6,241,132 (70.8)		
	Present	2,515,896 (28.9)	6,191,363 (71.1)		
Family history	Yes	1,516,774 (27.7)	3,966,295 (72.3)	6.1727	0.0130
	No	8,446,538 (24.5)	26,062,073 (75.5)		

**Table 2:** Multiple logistic regression analysis results about metabolic syndrome.

Variable	Level	Coefficients	Odds Ratio	95% CI	*p*-Value	${\mathrm{Point}}_{\mathrm{ij}}$
BMI	BMI < 25	0	1			0
	25 ≤ BMI < 30	1.81729	6.155	5.477–6.917	<0.0001	55
	30 ≤ BMI	3.27648	26.482	20.836–33.66	<0.0001	100
Marriage	Yes	0.38834	1.475	1.176–1.849	0.000829	12
	No	0	1			0
Employment	Yes	0	1			0
	No	0.20096	1.223	1.084–1.378	0.001099	6
Education	Low	0.39328	1.482	1.293–1.698	<0.0001	12
	High	0	1			0
Age	20–34	0	1			0
	35–64	1.41967	4.136	2.971–5.757	<0.0001	43
	65+	2.61083	13.61	9.363–19.785	<0.0001	80
Stroke	Yes	0.69463	2.003	1.37–2.928	0.00037	21
	No	0	1			0
Sex	Man	0.46728	1.596	1.116–2.282	0.010745	14
	Woman	0	1			0
Income	Lowest	0	1			7
	Low	−0.11727	0.889	0.772–1.025	0.105852	4
	High	−0.19741	0.821	0.709–0.951	0.008754	1
	Highest	−0.24427	0.783	0.671–0.915	0.002139	0
Smoking	Non	0	1			0
	Past	0.08861	1.093	0.932–1.281	0.274897	3
	Current	0.34904	1.418	1.196–1.681	<0.0001	11
Family history	Yes	0.32115	1.379	1.167–1.629	0.000183	10
	No	0	1			0
Age*Sex	35–64 & Man	0.0931	1.098	0.753–1.601	0.628911	28
	65+ & Man	−0.81425	0.443	0.293–0.671	0.000134	0
	otherwise (o.w.)	0	1			25

Likelihood Ratio Goodness of Fit Test: F = 223.34, *p*-value < 0.0001. Adjusted Wald Goodness of Fit Test: F = 118.57, *p*-value < 0.0001. Score Goodness of Fit Test: F = 110.03, *p*-value < 0.0001.

## Data Availability

The data that support the findings of this study are available from the Korea National Health and Nutrition Examination Survey.

## References

[1] Reaven G.M. (1988). Role of Insulin Resistance in Human Disease. Diabetes.

[2] Jung C.H., Park J.S., Lee W.Y., Kim S.W. (2002). Effects of smoking, alcohol, exercise, level of education, and family history on the metabolic syndrome in Korean adults. Korean J. Med..

[3] Lee W.Y., Park J.S., Noh S.Y., Rhee E.J., Kim S.W., Zimmet P.Z. (2004). Prevalence of the metabolic syndrome among 40,698 Korean metropolitan subjects. Diabetes Res. Clin. Pract..

[4] National Cholesterol Education Program (2001). Executive summary of the third report of the national cholesterol education program (NCEP) expert panel on detection, evaluation and treatment of high blood cholesterol in adults (adult treatment panel III). JAMA.

[5] Aguilar M., Bhuket T., Torres S., Liu B., Wong R.J. (2015). Prevalence of the metabolic syndrome in the United States, 2003–2012. JAMA.

[6] Tran B.T., Jeong B.Y., Oh J.K. (2017). The prevalence trend of metabolic syndrome and its components and risk factors in Korean adults: Results from the Korean National Health and Nutrition Examination Survey 2008–2013. BMC Public Health.

[7] Kim M.H., Lee S.H., Shin K.S., Son D.Y., Kim S.H., Joe H., Yoo B.W., Hong S.H., Cho C.Y., Shin H.S. (2020). The Change of Metabolic Syndrome Prevalence and Its Risk Factors in Korean Adults for Decade: Korea National Health and Nutrition Examination Survey for 2008–2017. Korean J. Fam. Pract..

[8] Yoo J.S., Jung J.I., Park C.G., Kang S.W., Ahn J.A. (2009). Impact of life style characteristics on prevalence risk of metabolic syndrome. J. Korean. Acad. Nurs..

[9] Iasonos A., Schrag D., Raj G.V., Panageas K.S. (2008). How to build and interpret a nomogram for cancer prognosis. J. Clin. Oncol..

[10] Mozina M., Demšar J., Kattan M., Zupan B. (2004). Nomogram for visualization of Naïve Bayesian classifier. Knowledge Discovery in Databases: PKDD 2004.

[11] Kim M.H., Seo J.H., Lee J.Y. (2019). A study on the method of constructing a nomogram for predicting dyslipidemia. Korean Data Inf. Sci. Soc..

[12] Kim M.H., Lee J.Y. (2020). How to construct a nomogram for hypertension using complex sampling data from Korean adults. Commun. Stat. Theory Methods.

[13] Korea Centers for Disease Control and Prevention (2018). The Seventh Korea National Health and Nutrition Examination Survey (KNHANES VII).

[14] Korean Statistical Information Service (KOSIS) Census, Statistic Korea, Republic of Korea. Https://kosis.kr/statis\ticsList/statisticsListIndex.do?menuld=M_01_01&vwcd=MT_ZTITLE&parmTabId=M_01_01&outLink=Y&entrType=#content-group.

[15] Chung S.M., Park J.C., Moon J.S., Lee J.Y. (2018). Novel nomogram for screening the risk of developing diabetes in a Korean population. Diabetes Res. Clin. Pract..

[16] Kshirsagar A.V., Chiu Y.L., Bomback A.S., August P.A., Viera A.J., Colindres R.E., Bang H. (2010). A hypertension risk score for middle-aged and older adults. J. Clin. Hypertens..

[17] Seo J.H., Kim H.J., Lee J.Y. (2020). Nomogram construction to predict dyslipidemia based on a logistic regression analysis. J. Appl. Stat..

[18] Rao J.N.K., Scott A.J. (1981). The analysis of categorical data from complex sample surveys: Chi-squared tests for goodness of fit the independence in two-way tables. J. Am. Stat. Assoc..

[19] Archer K.J., Lemeshow S., Hosmer D.W. (2007). Goodness-of-fit tests for logistic regression models when data are collected using a complex sampling design. Comput. Stat. Data Anal..

[20] Cassy S.R., Natário I., Martins M.R. (2016). Logistic regression modeling for complex survey data with an application for bed net use in Mozambique. Open J. Stat..

[21] Hosmer D.W., Lemeshow S., Sturdivant R.X. (2013). Applied Logistic Regression.

[22] Lee K.M., Kim W.J., Yun S.J. (2009). A clinical nomogram construction method using genetic algorithm and naïve Bayesian technique. J. Korean Inst. Intell. Syst..

[23] Park J.C., Kim M.H., Lee J.Y. (2018). Nomogram comparison conducted by logistic regression and naïve Bayesian classifier using type 2 diabetes mellitus. Korean J. Appl. Stat..

[24] Yang D. Build prognostic nomograms for risk assessment using SAS. Proceedings of the SAS Global Forum 2013.

[25] Akobeng A.K. (2007). Understanding diagnostic tests 3: Receiver operating characteristic curves. Acta Paediatr..

[26] Cook N.R. (2008). Statistical evaluation of prognostic versus diagnostic models: Beyond the ROC curve. Clin. Chem..

[27] D’Agostino R.B., Grundy S., Sullivan L.M., Wilson P., CHD Risk Prediction Group (2001). Validation of the Framingham coronary heart disease prediction scores: Results of a multiple ethnic group investigation. JAMA.

